# Prevalence and Classification of C-Shaped Canal and Radix in Mandibular Molars Using Cone-Beam Computed Tomography on Mexican Population

**DOI:** 10.3390/dj12070212

**Published:** 2024-07-10

**Authors:** Hugo Bojorquez Armenta, Angel Gustavo Romo Mireles, Javier Solis Martinez, Jesus Pescador Alvarez, Yarely Ramos Herrera, Omar Tremillo Maldonado, Oscar Almeda Ojeda, Jose Salas Pacheco, Gamaliel Ortiz Sarabia, Francisco Xavier Castellanos-Juarez, Sergio Salas Pacheco

**Affiliations:** 1 Faculty of Dentistry, Juarez University, Durango 34070, Mexico; endobojorquez@gmail.com (H.B.A.); angelgustavoromomireles@gmail.com (A.G.R.M.); javier.solis@ujed.mx (J.S.M.); jesus.pescador@ujed.mx (J.P.A.); dra.yarelyramos@hotmail.com (Y.R.H.); omar.tremillo@hotmail.com (O.T.M.); oscar.almeda@ujed.mx (O.A.O.); gamaliel.ortiz@ujed.mx (G.O.S.); 2 Institute for Scientific Research, Juarez University, Durango 34000, Mexico; jsalas_pacheco@hotmail.com (J.S.P.); xavier_castellanos@hotmail.com (F.X.C.-J.)

**Keywords:** cone-beam computed tomography, dental root, root canal preparation, root canal therapy

## Abstract

The diverse morphological configurations in teeth present clinical challenges in root canal treatment, complicating instrumentation and irrigation processes, which can lead to treatment failure. Understanding anatomical variations, such as C-shaped canals and radix entomolaris, enhances clinical skills and improves long-term endodontic treatment success rates. Cone-beam computed tomography (CBCT) offers superior diagnostic capabilities over conventional radiography, enabling the pre-operative detection of root configurations and canal numbers, facilitating personalized endodontic treatments. A total of 2173 teeth of a Mexican population, including 1057 first mandibular molars and 1116 s mandibular molars, were studied using only CBCT to identify C-shaped canals and radix configurations of patients who were treated from 2018 to 2023 at the Department of Radiology at the Faculty of Dentistry, Juarez University of the State of Durango, Mexico. C-shaped canals were identified in 160 teeth, with a prevalence of 0.2% in first mandibular molars and 14.1% in second mandibular molars. The highest frequency was in the left second mandibular molar (3.7) at 14.8%. Gender differences were significant, with higher prevalence in females (27.3%) compared to males (13.3%). The most common C-shaped canal configuration was type C2 (39.3%). Radix entomolaris was found in 52 teeth, with a prevalence of 3.4% in first mandibular molars and 1.4% in second mandibular molars. This research on a Mexican population using cone-beam computed tomography (CBCT) highlights significant findings in the prevalence and types of C-shaped canals and radix entomolaris in mandibular molars for this population. The left second mandibular molar (3.7) showed the highest prevalence at 14.8%, followed closely by the right second mandibular molar (4.7) at 13.5%, with a significant difference (*p* < 0.001). We found a significant difference in the prevalence of C-shaped canals between genders (*p* = 0.004, OR 1.78). Additionally, radix entomolaris (*p* < 0.001) was more frequently identified in first mandibular molars to a significant degree. These insights underscore the importance of CBCT in diagnosing complex root anatomies, which can greatly enhance the success rates of endodontic procedures by allowing for more tailored and precise treatments for this population.

## 1. Introduction

The method for achieving the objective of endodontic treatment is to completely clean and disinfect the root canals to eliminate all existing microorganisms [1]. Dentists face the challenge that teeth have various morphologies that can vary from the number to the arrangement of canals, which, if not efficiently treated, leads to treatment failure [2,3,4].

Cone-beam computed tomography (CBCT) has significantly advanced the field of dentistry by providing three-dimensional imaging with minimal radiation exposure. The most common clinical applications of CBCT include the treatment of root resorptions, surgical planning, and evaluation of outcomes; in addition, this technology has become fundamental for diagnosing complex anatomical variations, such as the C-shaped canal and radix, which present unique challenges during root canal treatment [5,6,7].

The C-shaped canal, most often found in second mandibular molars, was first described by Cooke and Cox in 1979 [8]. This poses a challenge for dentists, who must modify their treatment protocols to effectively clean and disinfect this type of canal. Due to the configuration of this morphological variation, certain angles may be difficult to reach or may not allow irrigation flow for cleaning the canals [9].

The probability of failure in endodontic treatment can reach up to 45% in dental organs with C-shaped canals [10], and its prevalence varies significantly, primarily depending on ethnicity. The global prevalence of C-shaped canals is 13.9%; China has the highest prevalence at 44.0%, while Brazil has the lowest at 6.8%. Mexico, on the other hand, has a prevalence of 14.2% [10]. Mexico is one of the most ethnically diverse countries, which is why different regions of the country generally exhibit very diverse genotypes and associated phenotypes [11].

Various researchers have attempted to classify the different configurations of C-shaped canals. Manning [12] and Melton [13] were the first to classify them, obtaining three classifications. Fans [14] expanded the classifications to five using micro-computed tomography, enhancing the precision and accuracy of its analysis. The most recent classification mentioned is as follows:

- Type 1 (C1): The C-shape is observed continuously without interruptions or separations.
- Type 2 (C2): The C-shape is observed interrupted, resembling a semicolon, but angles α and β are less than 60°.
- Type 3 (C3): Two or three separate canals are observed, with both angles α and β being less than 60°.
- Type 4 (C4): A single round or oval canal is observed in the cross-section.
- Type 5 (C5): No canal lumen is observed (it may only be visible near the apex).

Moreover, the supernumerary third root in a first mandibular molar, known as radix, was first described by Carabelli in 1844. This anatomical variation presents a different challenge for dentists during root canal treatment, as this third root is usually smaller than the other roots of the dental organ. It can be separated or partially fused with another root and often presents a pronounced curvature [15].

Generally, a mandibular molar presents with two roots. However, there is an anatomical variation where a third root is present; this supernumerary root is called radix [16]. To date, the origin of this morphological variation is not clear. The global prevalence of radix entomolaris is 5.6%, with the highest percentage found in China at 22.4% [17]. Carabelli was the first to report the presence of a supernumerary root, which he named radix entomolaris (RE) when it is located distolingually [15]. Carlsen and Alexandersen described an additional root found mesiobuccally as radix paramolaris (RP) [18].

While there is substantial research on the prevalence of C-shaped root canals and radix in various populations, there is limited data specifically focusing on the prevalence of different classifications of these anatomical variations. Many studies provide general prevalence rates without detailed breakdowns of the specific types or classifications of C-shaped canals. Most existing studies have been conducted on populations from specific regions. There is a lack of data on the Mexican population, which is known for its complex and diverse ethnic background. This could potentially reveal unique prevalence rates and classifications that differ from other studied populations. This research will allow for a comparative analysis with other populations using not only the general prevalence but also the different classifications, thereby contributing to a more comprehensive understanding of ethnic and regional differences in root canal morphology.

## 2. Materials and Methods

### 2.1. Study Design and Sample Collection

This is a retrospective, observational, and transversal study. RadiAnt Digital Imaging and Communications in Medicine (DICOM) viewer (Medixant, Poznan, Poland) files from 2018 to 2023, obtained with Newtom VGIEvo (Newtom, Imola, Italy), and patients who were born and reside in Durango City, Mexico, were included, covering every field of view. These files were subsequently analyzed by 3 specialists (endodontics, endoperiodontics, and oral radiology specialists) using RadiAnt software 2023.1 viewer (Medixant, Poznan, Poland). All subjects signed an informed consent agreement.

### 2.2. Ethics Approval

This study was submitted to and approved by the Ethics Committee of the Faculty of Medicine at Juarez University of the State of Durango, Mexico.

### 2.3. Statistical Analysis

IBM SPSS Statistics 25 software was utilized to determine measures of central tendency, dispersion, and frequency, and the measures were chi squared to establish significant differences.

### 2.4. Location

The study was conducted at the Research and Graduate Building of the Faculty of Dentistry, Juarez University of the State of Durango. The samples were obtained from the Department of Radiology at the Faculty of Dentistry, Juarez University of the State of Durango.

### 2.5. Dental Nomenclature

The Federation Dentaire Internationale (FDI) system of dental nomenclature was employed in this study.

### 2.6. Inclusion Criteria

Tomographic images with fully developed root formations were used.

### 2.7. Exclusion Criteria

- Teeth that have undergone root canal treatment.
- Retained, impacted, or embedded teeth.
- Teeth with developmental anomalies.
- Teeth with open apices.
- Calcified teeth or canal obliteration.
- Third molars.

### 2.8. Elimination Criteria

- Tomographic images with inadequate resolution.
- Tomographic images lacking patient data.

## 3. Results

**Figure 1:**
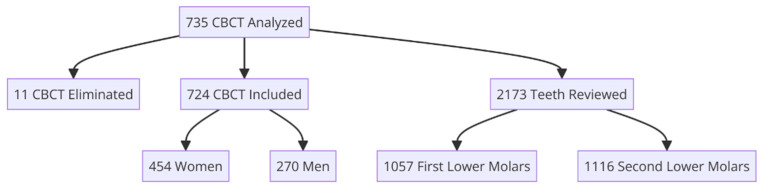
Flowchart of CBCT analysis and demographic distribution.

A total of 735 CBCT scans were obtained from January 2017 to December 2023, of which 724 CBCT scans were included in this research after applying the elimination criteria. The participants included 454 female patients and 270 male patients. The mean age was 44.15 years with a standard deviation of ±19.54. A total of 2173 teeth were studied, corresponding to 1057 first mandibular molars and 1116 s mandibular molars (Figure 1).

A total of 160 teeth with a C-shaped canal configuration were identified (Table 1). In first mandibular molars, a general frequency of 0.2% was observed, while in second mandibular molars, the general frequency was 14.1%. The left second mandibular molar (3.7) had the highest presence of C-shaped canals, with a frequency of 14.8%, followed by the right second mandibular molar (4.7), with 13.5%. The left and right first mandibular molars (3.6 and 4.6) had the lowest frequency, each with 0.2%.

Regarding gender, a significant difference was observed between the sexes, as the frequency of C-shaped canals was higher in women than in men (Table 2). Women exhibited a frequency of 27.3%, while men showed only 13.3%. Concerning the teeth studied (Table 3), in women, the left second mandibular molar (3.7) presented the highest frequency at 13.6%, followed by the right second mandibular molar (4.7) at 13.3%. The left and right first mandibular molars (3.6 and 4.6) each presented 0.2%. In men, the left second mandibular molar (3.7) had the highest frequency at 7.7%, followed by the right second mandibular molar (4.7) at 5.5%. There was no presence of C-shaped canals in the left and right first mandibular molars (3.6 and 4.6).

**Figure 2:**
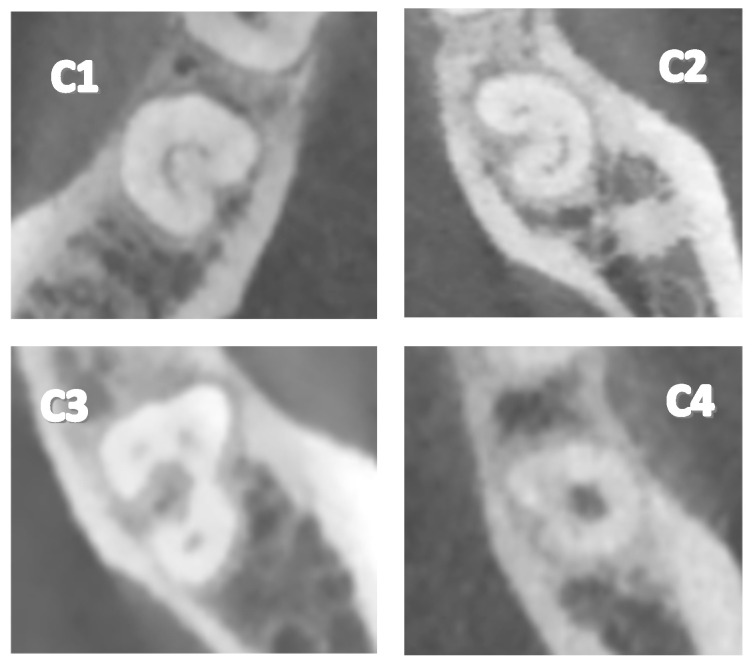
Types of C-shaped canal configurations in mandibular molars. (**C1**): The C-shape is continuous without interruptions or separations. (**C2**) The C-shape is interrupted, resembling a semicolon, with angles α and β less than 60°. (**C3**) Two or three separate canals are observed, with both angles α and β being less than 60°. (**C4**) A single round or oval canal is observed in the cross-section.

Regarding the different configurations of the C-shaped canal (Table 4, Figure 2), type C2 was identified most frequently at 39.3%. The other configurations were C1 at 26.3%, C3 at 18.8%, and C4 at 15.6%, while type C5 was not present in any cases. Concerning the teeth studied (Table 5), it was observed that in the left second mandibular molar (3.7), the most frequent configuration was C2 at 5.5%, followed by C1 at 4.3%, C3 at 2.7%, and C4 at 2.3%. In the right second mandibular molar (4.7), the most frequent configuration was C2 at 5.4%, C1 at 3.2%, C3 at 2.7%, and C4 at 2.2%. In the left and right first mandibular molars (3.6 and 4.6), only one case of C2 was present in each, resulting in a frequency of 0.2%.

The different configurations of C-shaped canals in relation to gender and teeth studied (Table 6) showed that in women, the left second mandibular molar (3.7) most frequently had the C2 configuration (6.2%), followed by the C1 type at 5.1%, the C3 type at 3.1%, and the C4 type at 3.1%. In the right second mandibular molar (4.7), the most frequent configuration was C2 at 6.6%, followed by C1 at 3.7%, C3 at 3.7%, and C4 at 3.1%.

A total of 52 teeth with the presence of radix were identified (Table 7). In first mandibular molars, a general frequency of 3.4% was observed, while in second mandibular molars, the general frequency was 1.4%. The right first mandibular molar (4.6) had the highest presence of radix with a frequency of 3.5%, followed by the left first mandibular molar (3.6) with a frequency of 3.3%. The right second mandibular molar (4.7) had a frequency of 2%, while the left second mandibular molar (3.7) had a frequency of only 0.9%.

Regarding gender, it was observed that men had a higher presence of radix compared to women (Table 8). Men exhibited a frequency of 7.7%, while women had a frequency of 7.0%, indicating a non-significant difference in the presence of radix. Concerning the teeth studied (Table 9), in women, the left first mandibular molar (3.6) had the highest frequency at 3.3%, followed by the right first mandibular molar (4.6) at 3.1%, the right second mandibular molar (4.7) at 1.7%, and the left second mandibular molar (3.7) at 1.4%. In men, the right first mandibular molar (4.6) had the highest frequency at 4.0%, followed by the left first mandibular molar (3.6) at 3.3%, the right second mandibular molar (4.7) at 1.7%, and the left second mandibular molar (3.7) at 0.4%.

**Figure 3:**
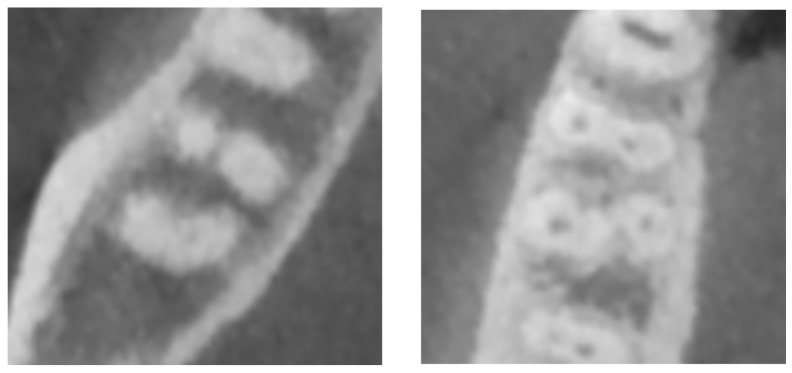
Types of radix variations in mandibular molars. Radix Entomolaris (RE): An accessory root located lingually (towards the tongue), most frequently found in the distolingual position. Radix Paramolaris (RP): An accessory root located buccally (towards the cheek), most frequently found in the mesiobuccal position.

Regarding the type of radix, an important difference was observed, with radix entomolaris having a frequency of 94.3%, and radix paramolaris having only 5.6% (Table 10, Figure 3). Concerning the teeth studied (Table 11), radix entomolaris was most frequently found in the left and right first mandibular molars (3.6 and 4.6) at 3.3%, while the right and left second mandibular molars (4.7 and 3.7) had frequencies of 1.6% and 1.1%, respectively. Radix paramolaris was only present in the right second mandibular molar (4.7) at 0.4% and the right first mandibular molar (4.6) at 0.2%.

The types of radix in relation to gender and the teeth studied (Table 12) showed that in women, radix entomolaris was most frequent in the left first mandibular molar (3.6) at 3.3%, followed by the right first mandibular molar (4.6) at 2.8%, and the left and right second mandibular molars (3.7 and 4.7) both at 1.4%. Radix paramolaris was only observed in the right second mandibular molar (4.7) with a frequency of 0.2%. In men, radix entomolaris had the highest frequency in the right first mandibular molar (4.6) at 4.0%, followed by the left first mandibular molar (3.6) at 3.3%, the right second mandibular molar (4.7) at 1.9%, and the left second mandibular molar (3.7) at 0.4%. Radix paramolaris was observed in the right first mandibular molar (4.6) at 0.3% and the right second mandibular molar (4.7) at 0.2%.

## 4. Discussion

Root canal treatment consists of a series of procedures designed to maintain or restore the health of periradicular tissues, thus fulfilling the biological objective of preventing and eliminating apical periodontitis [19,20]. A lack of knowledge about internal anatomy has previously been reported as the main cause of root canal treatment failure [21,22]. Karabucak and Bunes [4] reported an Odds Ratio (OR) of 4.38 for periapical lesions when a root canal was not treated. Similarly, Costa and Pacheco-Yanes [3] found an OR of 6.25, and Baruwa and Martins [23] found an OR of 3.1. An untreated canal could be the result of the operator’s limited knowledge of the tooth’s anatomy. However, dentists face anatomical variations resulting from genetic conditions, previous trauma, or diverse ethnicities [24,25]. One of the main variations in mandibular molars is radix entomolaris (an accessory lingual root) and paramolaris (an accessory buccal root) and the C-shaped canal.

The objective of our study focused on understanding the prevalence of these variations in a Mexican population. We analyzed 724 CBCT scans from the population, totaling 2173 mandibular molars (1057 first molars and 1116 s molars). We found the presence of C-shaped canals in 160 molars, corresponding to an overall frequency of 0.2% in first mandibular molars and 14.1% in second mandibular molars. This study has been repeated in various populations worldwide. Joshi and Shrestha [26] conducted a study on an Israeli population and found a global prevalence of C-shaped canals in first and second mandibular molars of 0.16% and 4.6%, respectively. In a Chinese population, the prevalence was 38.6% [19]. Martins and Mata [13] found a prevalence of C-shaped canal configurations of 0.6% and 8.5% in first and second mandibular molars, respectively, in a Portuguese population. In a Korean population, the prevalence was 32.7% [27]. These differences imply that genetic ancestry might be the most important factor in developing C-shaped canals and radix. The importance of conducting this study lies in the diverse prevalence rates found in each country, indicating that previously published studies may not be clinically relevant for Mexican dentists.

Our study also focused on analyzing the prevalence in each tooth. Interestingly, the left second mandibular molar (3.7) showed the highest prevalence at 14.8%, followed closely by the right second mandibular molar (4.7) at 13.5%. We found a significant difference (*p* < 0.001), indicating that the C-shaped canal is more likely to be found in 3.7 and 4.7. This significance is also maintained among C-Shaped canal classification (*p* < 0.001). This finding underscores the importance of conducting a thorough radiographic examination before initiating treatment, particularly in second mandibular molars.

To our knowledge, there are close to no studies showing the C-shaped root canal classification. In this sense, we found that C2 was the most prevalent of all, closely followed by C1, and the least prevalent was C5. Interestingly, this behavior is maintained regardless of the tooth.

We also divided our analysis by gender. Most studies report no differences between the sexes. Jannani et al., in 2018, found no significant differences in an Iranian cohort (*p* = 0.06), although a tendency toward significance can be observed [28]. Qian et al., in 2022, also reported no significant differences (*p* > 0.05) in a Chinese cohort [29].

We found significant differences (*p* = 0.004 OR 1.78 (1.18–2.68)) in the prevalence of C-shaped canals comparing both genders, with women exhibiting a higher frequency (17.8%) compared to men (10%), indicating that ethnicity might play an important role in the development of C-shaped canals.

Regarding radix, we identified 52 mandibular molars with this anatomical variation. The overall prevalence was 3.4% in first mandibular molars and 1.4% in second mandibular molars; we found a significant difference (*p* = 0.0264), indicating that radix has a higher likelihood to be found, with frequencies of 3.6 and 4.6. We found no significant difference among gender regarding radix (*p* = 0.788). Among radix types we found a significant difference (*p* < 0.001), indicating that entomolaris is the most common type of radix found in these teeth. A study on an Indian population [30] found a prevalence of 23%, and 32% in China, making these nations among those with the highest prevalence.

These anatomical variations highlight the importance of using advanced imaging techniques such as CBCT in endodontics. The early detection of C-shaped canals and radix can help customize treatment plans, ensuring thorough cleaning and disinfection. This approach can significantly improve the success rates of root canal treatments, particularly in teeth with complex root anatomies.

## 5. Conclusions

We report, to our knowledge, one of the first studies to include C-shaped root canal and radix types, and not only general prevalence. Our study found a notable prevalence of C-shaped canals in mandibular molars, with a higher frequency in second mandibular molars (14.1%) compared to first mandibular molars (0.2%). The left second mandibular molar (3.7) showed the highest prevalence at 14.8%, followed closely by the right second mandibular molar (4.7) at 13.5% with a significant difference (*p* < 0.001). We found a significant difference in the prevalence of C-shaped canals between genders (*p* = 0.004, OR 1.78), with women exhibiting a higher frequency (17.8%) compared to men (10%). This suggests that ethnicity and possibly gender-related genetic factors might influence the development of C-shaped canals. The presence of radix was significantly higher at 3.6 and 4.6 (*p* = 0.0264). Among the radix types, entomolaris was the most common (*p* < 0.001).

### Limitations of This Study

In this study, we used DICOM (Digital Imaging and Communications in Medicine) files, irrespective of their FOV. A standardized FOV, such as 5 × 5 cm, ensures consistent image quality and sufficient detail for accurate diagnosis and treatment planning. The Mexican population is ethnically diverse, and each state has its own almost exclusive ancestry. This study only included patients from Durango, Mexico. Studies from other states or a study including all states are suggested to establish a better understanding of our findings

## Figures and Tables

**Table 1:** Prevalence of C-shaped canal.

	3.7	4.7	3.6	4.6	*p* *
n	560	556	539	518	<0.001
Absence	85.2%	86.5%	99.8%	99.8%	
Presence	14.8%	13.5%	0.2%	0.2%	

* *p* was calculated by chi square. The FDI system was used for nomenclature.

**Table 2:** Gender differences in the prevalence of C-shaped canal.

	Women (454)	Men (270)	*p* *	OR **
Absence	82.15%	90%	0.004	1.78 (1.18–2.68)
Presence	17.8%	10%		

* *p* was calculated by chi square. ** Odds Ratio was used to establish risk.

**Table 3:** Presence of C-shaped canal by gender.

	3.7		*p* *	4.7		*p* *	3.6		*p* *	4.6		*p* *
	Women	Men		Women	Men		Women	Men		Women	Men	
Absence	86.3%	92.2%	0.016	86.7%	94.4%	0.001	99.7%	100%	0.998	99.7%	100%	0.993
Presence	13.6%	7.7%		13.2%	5.5%		0.2%	0%		0.2%	0%	

* *p* was calculated by chi square.

**Table 4:** Frequency of different C-shaped canal configurations.

Type	N° of Teeth	Percentage
C1	42	26.3%
C2	63	39.3%
C3	30	18.8%
C4	25	15.6%
C5	0	0%

**Table 5:** Frequency of C-shaped canal configurations.

	3.7	4.7	3.6	4.6	*p* *
	n = 560	n = 556	n = 539	n = 518	<0.001
Absence	85.2%	86.5%	99.8%	99.8%	
C1	4.3%	3.2%	0%	0%	
C2	5.5%	5.4%	0.2%	0.2%	
C3	2.7%	2.7%	0%	0%	
C4	2.3%	2.2%	0%	0%	
C5	0%	0%	0%	0%	

* *p* was calculated by chi square.

**Table 6:** Frequency of C-shaped canal by gender.

	3.7			4.7			3.6		4.6	
	Women (352)	Men (208)	*p* * 0.171	Women (346)	Men (210)	*p* * 0.015	Women (332)	Men (207)	Women (319)	Men (199)
Absence	82.3%	89.9%		82.6%	92.8%		99.6%	100%	99.6%	100%
C1	5.1%	2.8%		3.7%	2.3%		0%	0%	0%	0%
C2	6.2%	4.3%		6.6%	3.3%		0.4%	0%	0.4%	0%
C3	3.1%	1.9%		3.7%	0.9%		0%	0%	0%	0%
C4	3.1%	0.9%		3.1%	0.4%		0%	0%	0%	0%
C5	0%	0%		0%	0%		0%	0%	0%	0%

* *p* was calculated by chi square.

**Table 7:** Prevalence of radix in mandibular molars.

	3.7 (559)	4.7 (556)	3.6 (539)	4.6 (518)	*p* *
Absence	99.1%	98%	96.7%	96.5%	0.0264
Presence	0.9%	2%	3.3%	3.5%	

* *p* was calculated by chi square.

**Table 8:** Gender differences in the prevalence of radix.

	Women (454)	Men (270)	*p* *
Absence	92.9%	92.2%	0.788
Presence	7.0%	7.7%	

* *p* was calculated by chi square.

**Table 9:** Prevalence of radix in mandibular molars by gender.

	3.7		4.7		3.6		4.6	
	Women (352)	Men (208)	Women (346)	Men (210)	Women (332)	Men (207)	Women (319)	Men (199)
Absence	98.5%	99.5%	98.2%	97.6%	96.6%	96.6%	96.8%	95.9%
Presence	1.4%	0.4%	1.7%	2.3%	3.3%	3.3%	3.1%	4.0%

**Table 10:** Frequency of radix types (entomolaris vs. paramolaris).

Type	Number of Teeth	Percentage	*p* *
Entomolaris	50	94.3%	<0.001
Paramolaris	3	5.6%	

* *p* was calculated by chi square.

**Table 11:** Prevalence of radix types.

	3.7 (560)	4.7 (556)	3.6 (539)	4.6 (518)	*p* *
Absence	98.9%	98%	96.7%	96.5%	0.1657
Entomolaris	1.1%	1.6%	3.3%	3.3%	
Paramolaris	0%	0.4%	0%	0.2%	

* *p* was calculated by chi square.

**Table 12:** Frequency of radix types by gender.

	3.7		*p* *	4.7		*p* *	3.6		4.6	
	Women (553)	Men (208)	0.42	Women (346)	Men (210)	0.57	Women (332)	Men (207)	Women (319)	Men (199)
Absence	98.5%	99.8%		98.2%	97.6%		96.6%	96.6%	96.8%	95.9%
Entomolaris	1.4%	0.4%		1.4%	1.9%		3.3%	3.3%	2.8%	4%
Paramolaris	0%	0%		0.2%	0.4%		0%	0%	0.3%	0%

* *p* was calculated by chi square.

## Data Availability

The data presented in this study are available on request from the corresponding author due to privacy and legal reasons.
